# A case of catatonia in the aftermath of the COVID-19 pandemic: does autism spectrum matter?

**DOI:** 10.1186/s12991-021-00377-9

**Published:** 2021-12-16

**Authors:** Liliana Dell’Osso, Giulia Amatori, Camilla Gesi, Claudia Carmassi

**Affiliations:** 1 Department of Clinical and Experimental Medicine, University of Pisa, Pisa, Italy; 2 Department of Mental Health and Addiction, ASST Fatebenefratelli-Sacco, Milan, Italy

**Keywords:** Catatonia, Autism spectrum disorder (ASD), COVID-19, Rumination, Neurodevelopmental disorder, Electroconvulsive therapy (ECT)

## Abstract

**Background:**

There is growing concern about the psychopathological consequences of the COVID-19 pandemic. The prolonged stress due to the spreading fear of the contagion and to the enforced containment measures are deemed to trigger recurrences of preexisting mental disorders as well as the onset of new ones. From such perspective, clinical cases may be of primary ground to identify individual features and pandemic-related factors predisposing to the development of serious psychiatric symptoms.

**Case presentation:**

Mr. R. is a 64-year-old, married, unemployed man, whose premorbid personality was characterized by relevant autistic traits. The patient developed catatonia in the context of the COVID-19 pandemic. We aimed at discussing the role of both preexisting and precipitating factors.

**Conclusions:**

Autism spectrum could represent a predisposing factor for severe psychopathological outcome and catatonia. Furthermore, the present clinical case highlights the role of COVID-19 pandemic in influencing physical and mental health.

## Background

Growing attention is being devoted to mental health outcomes in the aftermath of COVID-19 pandemic [1, 2], which is associated with levels of psychological distress that, in a number of cases, reach the threshold of clinical relevance. According to a recent systematic review, high rates of anxiety, depression, post-traumatic stress disorder, and psychological distress were found in the general population during the COVID-19 pandemic in China, Spain, Italy, Iran, USA, Turkey, Nepal and Denmark [3–5]. Risk factors for the development of psychiatric symptoms included female gender, young age (≤ 40 years), unemployment, student status, and frequent exposure to social media and news about the COVID-19 pandemic [3]. People with established mental illnesses and/or predisposing traits are at higher risk too [3] and they may be especially vulnerable to persisting fear of contagion, measures of social distancing and disruption of healthcare services. We present a case of catatonia, developed in the context of the COVID-19 pandemic, discussing the role of predisposing autism spectrum traits and that of precipitating factors related to the pandemic.

## Case presentation

Mr. R. is a 64-year-old, married, unemployed man, without a family history of mental disorders. He lives with his wife and has no children. His relatives describe him as an introverted person, with emphatic and social difficulties, a rigid adherence to routine, restricted interests and behaviors, always struggling with anxiety and with fear of economic strain. After a high school diploma, he entered the Law school and despite being an excellent student he had to quit university due to financial reasons. He was then employed in the family firm where, in the context of an increase in personal and work responsibilities, his strong adherence to routine and tendency to social isolation progressively intensified. At the age of 36, when he was the managing director, the firm went bankrupt and Mr. R. began to suffer from sporadic panic attacks. From then on, despite obtaining a stable job as a bank clerk, he was deeply preoccupied with the fear of losing everything and undergoing financial strain. While never delusional, the rumination about economic stability was pervasive and associated with frequent mood swings in both polarities and somatic symptoms without organic cause (mainly epigastric pain and dysphonia). However, he always refused to seek for psychiatric help and showed progressive social withdrawal. Since COVID-19 began spreading through Italy, Mr. R has experienced severe isolation, struggling to work and spending most of the time at home alone and talking to himself. The enforced lock-down measures further discouraged Mr. R. and his wife to seek for help.

In March 2021, the clinical picture definitely deteriorated as Mr. R. developed a deep rumination focused on the fear of contracting the COVID-19 (in spite of repeated negative swab tests) progressively increasing to racing thoughts and delusional hypochondria, together with hyporexia and severely disrupted circadian rhythms. When convinced to see a psychiatrist, Mr. R. presented in a full-blown catatonic state, with stupor, posturing, negativism, waxy flexibility, mutacism, lack of facial expressiveness, fixed gaze, echolalia and occasional episodes of agitation, a clinical picture that meets the criteria for the diagnosis of catatonia according to DSM-5.

After inpatient admission, Mr. R. was treated with increasing doses of lorazepam (up to 10 mg/day). After a few of days with no improvement, biweekly electroconvulsive therapy (ECT) was initiated. Mr. R. showed improvement in motor signs and verbal activity since the first session, and fully recovered after completion of the eight-session ECT treatment. Upon discharge, after 4 weeks, Mr. R was prescribed olanzapine 20 mg/day, paroxetine 10 mg/day and lorazepam 2 mg/day as maintenance therapy and administered the Structured Clinical Interview for DSM-5 Disorders (SCID-5), the Autism-Spectrum Quotient (AQ) [6] and the Adult Autism Subthreshold Spectrum (AdAS Spectrum) [7]. He reported a diagnosis of bipolar disorder with catatonic features, with comorbid panic disorder and a clinically significant level of autistic traits [8] (see Table 1). The patient also gave written informed consent to the processing of health data for research purposes.

**Table 1:** Autism-Spectrum Quotient (AQ) and Adult Autism Subthreshold Spectrum (AdAS Spectrum) scores obtained by Mr R

Autism-Spectrum Quotient (AQ)	Scores	AdAS spectrum	Scores
Social skill	6/10	Childhood/adolescence	8/21
Attention switching	7/10	Verbal communication	10/18
Attention to detail	5/10	Non-verbal communication	15/28
Communication	7/10	Empathy	2/12
Imagination	5/10	Inflexibility and adherence to routine	20/43
		Restricted interests and rumination	15/21
AQ total score	30/50	Hyper-/hyporeactivity to sensory input	10/17
		AdAS Spectrum total score	80/160

## Discussion and conclusions

Catatonia is a potentially life-threatening, motor syndrome, characterized by mental and physical immobilization and stereotypic behaviors, which may alternate with agitation and complicated with rigidity, hyperthermia and autonomic dysfunction. The mainstay of treatment for established catatonia is benzodiazepines and electroconvulsive therapy (ECT) [9]. Furthermore, antipsychotics discontinuation—especially first-generation ones—is recommended for the treatment of catatonia [10]. Conversely, it has been pointed out that the prevalence of catatonia in association with schizophrenia has decreased markedly after the introduction of antipsychotic agents, across the 1950s, increasing the percentage of cases associated with mood disorders or general medical conditions. Furthermore, in the early twentieth century, catatonia has progressively been considered a common pathway for many severe mental disorders [11, 12], occurring in a broad variety of diseases as a sort of end point. Recent literature, for instance, has shown that catatonia is not uncommon among people with autism spectrum disorder (ASD).

In a recent study, catatonia was found in 18% of adolescents admitted to a psychiatric ward because of a variety of mental disorders, including pervasive developmental disorder [13]. In two prevalence studies [14] catatonia was found in 12–17% of a large sample of adolescents and young adults with ASD. Furthermore, a recent review highlighted how the use of ECT, useful for the treatment of catatonia, can markedly reduce self-injuring behaviors in subjects suffering from ASD or mental disability [15]. Therefore, a putative role of ASD as a risk factor for catatonia is worth of further studies. On the other hand, a growing literature supports the existence of a broad autism spectrum [16–25], devoid the typical functional impairment of severe forms, but in phenomenological and etiological continuity with the ASD.

In this context, the autistic spectrum of Mr. R., albeit under the clinical threshold, could have been a ground of vulnerability for the development of catatonia, coupled with overwhelming COVID-19-related stress and with delayed antipsychotic treatment. The COVID-19 pandemic posed indeed a significant burden on physical and mental health, social support, job opportunities, economic condition and even primary necessities. Fear of contagion and enforced social distancing affected mental health services worldwide. Several outpatient services were halted, as most of resources were diverted to the management of urgent cases or to the treatment of COVID-19 infection, leading to an overall increase of psychiatric disorders. A number of reports have been recently published describing cases of catatonia sprouting during the pandemic [26–32] and focusing on the use of ECT in the treatment of neuropsychiatric symptoms associated with COVID-19 [33]. While most of them discuss the direct or immune-mediated effect of COVID-19 on peripheral and central nervous system impairments, a handful of studies focus instead on the role of pandemic-related stressors (enforced self-isolation, changes to usual routines, financial strain) as precipitating factors for new-onset or recurring mental illnesses leading to catatonia, especially among people with predisposing traits. Based on the case of Mr. R., we highlight a third possible contributing factor, which relates to the late referral and delayed treatment during the prolonged state of emergency befalling to healthcare around the world. We hypothesize that the unprecedented social distancing, movement restriction, and disruption to healthcare due to COVID-19 might have jeopardized regular access to mental health services and hindered treatment adherence, making patients vulnerable to recurrences and prone to display poorer outcomes. Indeed, catatonic states often represent a final, common pathway of severe, untreated mental illnesses, as suggested by the sharp decrease in the prevalence of catatonic schizophrenia in the 1950s, after the neuroleptics’ advent provided the very first tool affecting the natural history of psychotic spectrum disorders [10].

In conclusion, the role of autism spectrum as predisposing factor for severe psychopathological outcome [34] and to catatonia warrants further investigations. Conversely, the pandemic provides a unique opportunity to observe psychosocial mechanisms of resistance, vulnerability and resilience.

## Data Availability

The datasets analyzed during the current study are available from the corresponding author on reasonable request.
